# Computer-Assisted Microcatheter Shaping for Intracranial Aneurysm Embolization

**DOI:** 10.3390/brainsci13091273

**Published:** 2023-08-31

**Authors:** Heng Yang, Liquan Xu, Yanjiang Li, Hanqiang Jiang, Wei Ni, Yuxiang Gu

**Affiliations:** 1 Department of Neurosurgery, Huashan Hospital, Fudan University, Shanghai 200040, China; yangheng901109@163.com (H.Y.); xuliquan2858@sohu.com (L.X.); lyj113131275@163.com (Y.L.); jhq333000@163.com (H.J.); guyuxiang1972@126.com (Y.G.); 2 Neurosurgical Institute, Fudan University, Shanghai 200040, China; 3 Shanghai Clinical Medical Center of Neurosurgery, Shanghai 200040, China; 4 National Center for Neurological Disorders, Huashan Hospital, Shanghai Medical College, Fudan University, Shanghai 200040, China

**Keywords:** microcatheter shaping, intracranial aneurysm, computer-assisted

## Abstract

Background: This study investigates the accuracy, stability, and safety of computer-assisted microcatheter shaping for intracranial aneurysm coiling. Methods: Using the solid model, a microcatheter was shaped using computer-assisted techniques or manually to investigate the accuracy and delivery of microcatheter-shaping techniques in aneurysm embolization. Then, forty-eight patients were randomly assigned to the computer-assisted microcatheter-shaping (CAMS) group or the manual microcatheter-shaping (MMS) group, and the accuracy, stability, and safety of microcatheter in the patients were compared between the CAMS and MMS groups. Results: The speed of the successful microcatheter position was significantly faster in the CAMS group than in the MMS group (114.4 ± 23.99 s vs. 201.9 ± 24.54 s, *p* = 0.015) in vitro. In particular for inexperienced operators, the speed of the microcatheter position with the assistance of computer software is much faster than manual microcatheter shaping (93.6 ± 29.23 s vs. 228.9 ± 31.27 s, *p* = 0.005). In vivo, the time of the microcatheter position in the MMS group was significantly longer than that in the CAMS group (5.16 ± 0.46 min vs. 2.48 ± 0.32 min, *p* = 0.0001). However, the mRS score at discharge, the 6-month follow-up, and aneurysm regrowth at the 6-month follow-up were all similar between the groups. Conclusions: Computer-assisted microcatheter shaping is a novel and safe method for microcatheter shaping that introduces higher accuracy in microcatheter shaping during the treatment of intracranial aneurysms. Significant: Endovascular coiling of intracranial aneurysms can be truly revolutionized through computer assistance, which could improve the endovascular treatment of aneurysms.

## 1. Introduction

Endovascular neurosurgery is a rapidly evolving field, with the continuous development of techniques and technologies [1]. Endovascular techniques for the treatment of intracranial aneurysms have evolved at an especially remarkable pace [2]. Detachable endovascular coiling was originally conceived by Guglielmi as a major milestone in the treatment of intracranial aneurysms. Ever since, coil technology, stent manufacturing, and innovations in navigation materials have been advanced [3]. Endovascular treatment has mostly replaced traditional open surgery and has revolutionized the treatment of intracranial aneurysms. This trend in aneurysm management techniques has significantly transformed conventional training for neurosurgeons.

Safe and efficient microcatheter navigation within the parent vessel and aneurysmal sac during aneurysm coiling is crucial to avoid complications, such as aneurysm perforation or thrombosis. Stable and precise microcatheter shaping is fundamental to successful embolization. The convenience and safety of microcatheterization depend on the aneurysmal anatomy and the experience of the neurosurgeons. Microcatheter tips are traditionally shaped manually by the operators to achieve smooth navigation and stable packing of the coil into the aneurysmal sac; the modifications are guided using digital subtraction angiography (DSA)-based three-dimensional (3D) anatomy of the aneurysm and parent vessel. However, this shaping procedure is remarkably challenging for junior neurosurgeons with limited experience. Therefore, novel shaping methods to improve aneurysm accessibility, especially those at uncommon locations, are needed to ensure efficiency and reduce the risk of complications during the procedure. In addition, a specific coil-shaping methodology can improve intra-aneurysmal microcatheter positioning during an aneurysm embolization.

Recent advancements in computer-based calculation software have facilitated computational assistance in several scientific and medical fields, including the neuro-interventional field [4,5]. These advances have substantially improved the anatomical understanding of aneurysms and parent vessels. Data from endovascular treatments of intracranial aneurysms are being used to derive a direct algorithm for microcatheter shaping and access routes.

Considering the above-mentioned theories and techniques, we believe that endovascular coiling of intracranial aneurysms can be truly revolutionized through computer assistance. We hypothesized that using 3D image parameter-analyzing algorithms to guide microcatheter shaping could improve the accuracy and stability of endovascular coils used during the treatment of intracranial aneurysms.

## 2. Methods

### 2.1. Study Design

To investigate the efficacy of the computer-assisted microcatheter-shaping technique on the microcatheter positioning for different seniority physicians, microcatheter shaping was performed by either experienced or inexperienced operators. This study used two datasets. Dataset 1 included 10 selected patients with intracranial aneurysms from January 2015 to June 2015 to construct hollow elastic models for an in vitro prospective controlled study. Dataset 2 enrolled consecutive patients with intracranial aneurysms from September 2021 to December 2021 who received endovascular treatment for an in vivo prospective randomized study. All patients were blinded to these cases. Patients were included if they presented with an unruptured intracranial saccular aneurysm, were aged between 18 and 75 [6,7], had an aneurysm size between 3 and 10 mm in diameter [8,9,10], and consented to participate in this trial. Participants were excluded if they had an aneurysm embolized with a flow diverter. The local medical research ethics committee approved the experimental protocol, and written informed consent was obtained from all participants.

### 2.2. In Vitro Study

#### 2.2.1. Hollow Elastic Model Preparation

The 3D image data, including the aneurysm and parent artery, were loaded into a 3D measurement software (UKnow, Qianglian Zhichuang (Beijing) Technology Co., Ltd., Beijing, China) in the DICOM format of 3D-DSA. The aneurysms of interest and their corresponding parent vessels were interactively 3D-segmented using the image thresholding method [11,12]. The surfaces of segmented aneurysms and parent vessels were extracted and converted into standard STL files. Subsequently, a 3D printer was used to print the model with a flexible, transparent, and photosensitive resin. A silicone coating was added to the outer layer of the model using the membrane penetration technique. The inner cavity was printed at a ratio of 1:1 to the true vascular cavity.

#### 2.2.2. Grouping of the Models

The same 10 aneurysm models were allocated to four operators, respectively (*n* = 10/group), including two experienced operators (case volume > 40 per year) and two inexperienced operators (case volume < 10 per year). Within each group, computer-generated randomization was used to determine whether the microcatheter (Echelon 10, pre-shaped 45° tip, Medtronic, Minneapolis, MN, USA) was shaped using computer-assisted techniques or manually.

#### 2.2.3. Computer-Assisted Shaping Technique

**Figure 1:**
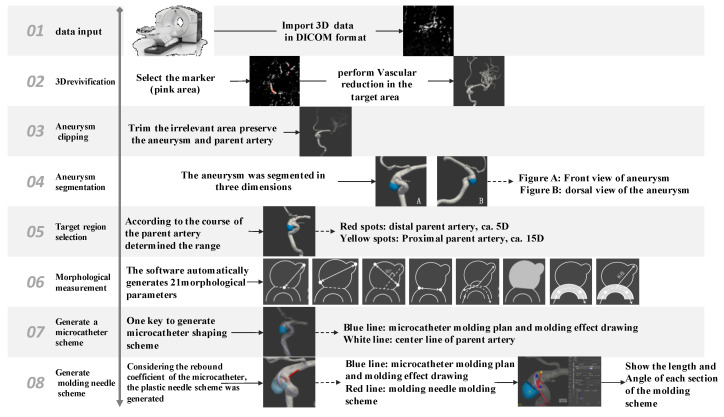
Shaping Technology Roadmap.

The flow chart of the microcatheter path computation is in Figure 1. After three-dimensional (3D) angiography of the aneurysm was performed, the 3D imaging digital images and communications in medicine (DICOM) data were transferred from the workstation to the software. Segmentation and automatic measurement of the aneurysm’s morphological parameters (including the diameter of the neck, aneurysm height, width, volume, and inflow angle) were conducted before the intelligent calculation of the microcatheter path.

**Figure 2:**
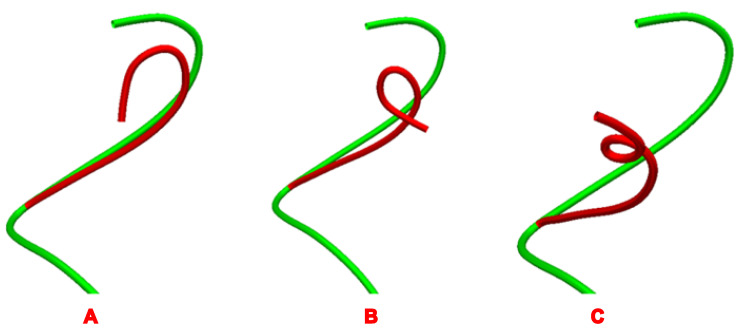
Shape calculation by resilience coefficients; (**A**) 1.2, (**B**) 2.0, (**C**) 3.0.

Next, an algorithm was performed inversely to start from the aneurysm and end at the distal vessel to accurately place the tip of the microcatheter at the center of the aneurysm. The simulation was implemented based on collision detection and the centerline constraint algorithm. Collision points against the vessel walls were mathematically detected to generate the initial microcatheter path. It was corrected by centerline constraint formulation to conform it to the shape and direction of the vessel and not to extend the vessel wall. Afterward, the microcatheter type was inputted into the software to generate rebound coefficients (Figure 2). Finally, the appropriate microcatheter path was established, with the length and angle of each segment of this route as an automatic output of the algorithm. The shaping mandrel and microcatheter were molded as shown in the microcatheter-shaping method of the MMS group.

#### 2.2.4. Simulation Experiment

**Figure 3:**
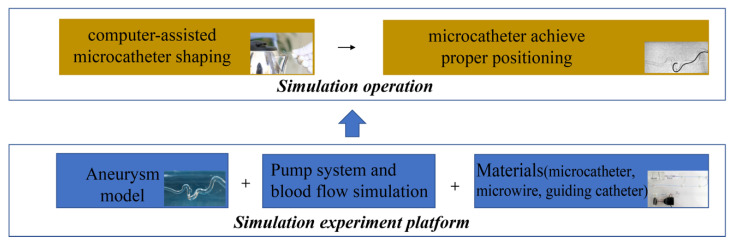
In vitro study. The aneurysm model, pump system, and interventional materials constituted the platform of the in vitro experiment, and a doctor conducted the microcatheter shaping according to the shaping plan. Then, the microcatheter was navigated into the hollow elastic aneurysm models with or without microwire guiding.

On the platform of the in vitro experiment, red ink was infused and continuously circulated inside the model to simulate the blood flow with the help of a water pump. The lumen was clearly visible using a video camera and a monitor. The microcatheter was pre-plastically processed and then operated on to enter the hollow elastic aneurysm models with or without microwire guidance, and the accuracy and time of the microcapsule were recorded. A microwire (Transcend 14 Soft Tip, Stryker, Neurovascular, Fremont, CA, USA) could be used for navigation if there were common patient-related technical difficulties, including anatomic variations in the aortic arch, stenosis, or serious tortuosity of the internal carotid artery (ICA) and vertebral artery, which can prevent the advancement of the guiding catheter [13]. A camera was used to record the entire procedure until appropriate positioning of the microcatheter was achieved. (Figure 3).

### 2.3. In Vivo Study

From September 2021 to December 2021, 59 subjects were screened at Huashan Hospital, Fudan University; 11 subjects were excluded based on the exclusion criteria, and 48 met the inclusion criteria. The included subjects were assigned to either the computer-assisted microcatheter-shaping (CAMS) group or the manual microcatheter-shaping (MMS) group (*n* = 24/group).

### 2.4. Endovascular Treatment

Endovascular coiling treatment has been previously described [14,15]. In the MMS group, the operator manually shaped the tip of the microcatheter by shaping the mandrel and steam based on their analysis of the 3D rotational DSA images of the aneurysms. In the CAMS group, the microcatheter tip was shaped by the operators according to the mandrel guiding images provided by the computer based on algorithmic analysis of the DSA images of the aneurysms. Adjunctive coiling techniques, such as balloon remodeling, stenting, and double microcatheters, were used as required. The coils were selected according to the surgeon’s preference. Microcatheter delivery was classified according to the following standards: excellent, the microcatheter enters the aneurysm sac successfully without a microwire; moderate, the microcatheter enters the aneurysm sac successfully with a microwire; poor, the microcatheter failed to guide into the aneurysm sac or the microcatheter needs to be reshaped.

### 2.5. Trial Outcomes

For both in vivo and in vitro studies, the primary outcome was the time of successful microcatheter positioning. Successful positioning was defined as achieving a distance between the microcatheter tip and the anatomic center of the aneurysms within 1/4 of the length of the aneurysm, which was confirmed by 3D rotational angiography. The timing was started when the microcatheter was removed from the end of the guiding catheter.

Secondary outcomes were the mRS scores at discharge and the 6-month follow-up and aneurysm regrowth at the 6-month follow-up.

Safety variables included any intraoperative hemorrhagic or ischemic complications during the procedure. If neurological deterioration developed, additional neuroimaging was required. Neurosurgeons were unblinded to the treatment group assignments and reported any serious adverse events on our web-based database.

The stability of the microcatheter was assessed at the end of the procedure. The microcatheter was evaluated as stable if the coiling was completed without premature prolapse [16].

### 2.6. Statistical Analysis

All analyses were performed on the intention-to-treat population using SPSS 22. Categorical variables were compared between groups using Fisher’s exact test. Continuous variables were compared using a *t*-test, except in cases where medians and interquartile ranges were reported, where a Wilcoxon rank sum test was performed. The following clinical outcomes were included as dependent variables in separate models: mRS of 0–2 (good outcomes), mRS of 3–4 (moderate outcomes), and mRS of 5–6 (poor outcomes).

## 3. Results

### 3.1. Accuracy and Stability of Microcatheter Shaping in the Aneurysm Model

A total of 40 microcatheters were shaped either by computer-assisted techniques or manually (*n* = 20 in each group) for microcatheter insertion in the aneurysm models. All the aneurysms were in the distal section of the intracranial artery (ICA) or the anterior communicating artery. The comparison of different microcatheter-shaping methods showed that the position speed in the CAMS group was significantly faster than that in the MMS group (114.4 ± 23.99 s vs. 201.9 ± 24.54 s, *p* = 0.015). However, no significant difference in microcatheter delivery was detected between the groups (*p* = 0.52; Table 1).

The subgroup analysis of the accuracy of the microcatheter position and the microcatheter delivery by experienced and inexperienced operators is provided in Table 2. Computer-assisted techniques significantly improved the speed of the microcatheter position for inexperienced operators compared with the manual microcatheter shaping (93.6 ± 29.23 s vs. 228.9 ± 31.27 s, *p* = 0.005). However, these differences were not significant for experienced operators in the groups.

### 3.2. Accuracy, Stability, and Safety of Computer-Assisted Microcatheter Shaping in Patients

Case presentation, treatment, and outcome details were assessed to confirm further the accuracy, stability, and safety of computer-assisted microcatheter shaping for aneurysm coiling in comparison to manual shaping. The patient and aneurysm characteristics, treatment details, and anatomical and clinical outcomes are shown in Table 3. A total of 48 patients underwent endovascular embolization, including 24 in the CAMS group and 24 in the MMS group. No significant differences in the baseline characteristics were found between the groups. All aneurysms were fully embolized except for one that was partially embolized in the CAMS group. Moreover, no perioperative complications were detected among the cases. In vivo, the microcatheter position time in the MMS group was significantly longer than that in the CAMS group (5.16 ± 0.46 min vs. 2.48 ± 0.32 min, *p* = 0.0001). However, the accuracy of the microcatheter position and stability was similar between the groups. Meanwhile, the mRS at discharge and aneurysm regrowth at the 6-month follow-up were similar between the groups (Table 4).

### 3.3. Illustrative Cases

**Figure 4:**
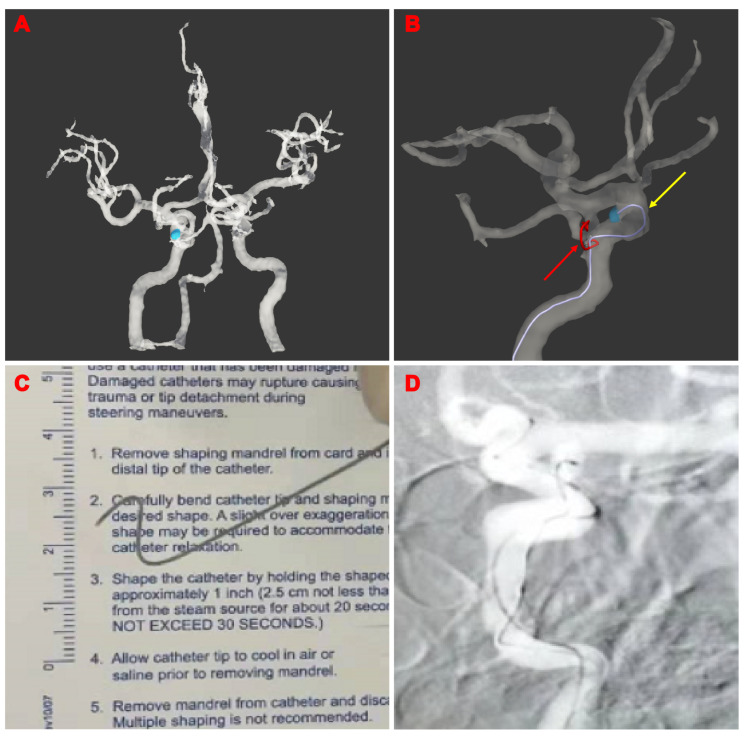
Illustrative Cases 27: A male patient in their 40s with an incidental 5 mm left ICA ophthalmic aneurysm. (**A**) Three-dimensional reconstruction of the aneurysms; (**B**) shaping plan of the microcatheter. The yellow arrow is the shaping plan of the microcatheter, and the red arrow is the shaping plan of the needle; (**C**) microcatheter pre-shaping completed; (**D**) successful microcatheter guidance into the aneurysm.

Patient 27: A 46-year-old male patient with an incidental 5 mm left ICA ophthalmic aneurysm was embolized with the computer-assisted technique. The information on the aneurysm and parent artery was targeted and extracted with UKnow^®️^. The optimal route for the microcatheter was then simulated and shaped. Subsequently, the microcatheter was positioned to embolize the aneurysm with stent-assisted coiling in approximately 2 min (Figure 4).

**Figure 5:**
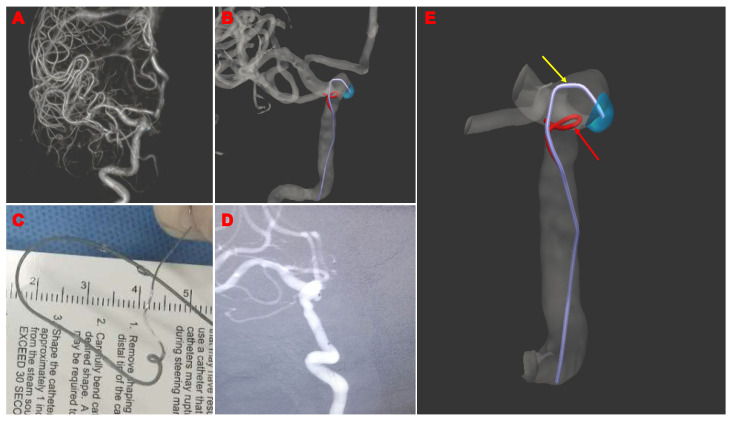
Illustrative Cases 39: A female patient in their 40s with an incidental 3.9 mm left ICA ophthalmic aneurysm. (**A**) Three-dimensional reconstruction of aneurysms; (**B**) shaping plan of the microcatheter; (**C**) microcatheter pre-shaping completed; (**D**) the microcatheter is successfully guided into the aneurysm; (**E**) the yellow arrow is the shaping plan of the microcatheter and the red arrow is the shaping plan of the needle.

Patient 39: A woman in her 40s with a left ICA ophthalmic aneurysm was embolized using the computer-assisted technique. The information on the aneurysm and parent artery was targeted and extracted with UKnow^®️^. The optimal route for the microcatheter was then simulated and shaped. The microcatheter is successfully guided into the aneurysm within approximately 2 min (Figure 5).

## 4. Discussion

Precise microcatheter navigation is an essential factor for the success of endovascular treatment of cerebral aneurysms. Although the use of an intermediate catheter increased the success rate of microcatheter navigation in a case of difficult vascular access anatomy [17], optimized microcatheter shaping can guarantee its accuracy and stability. Therefore, microcatheter shaping is a fundamental technique in aneurysm embolization. Owing to the various anatomic characteristics of aneurysms and arterial paths to the aneurysms, effective microcatheter shaping requires rich experience and technical expertise. In our study, computer-assisted microcatheter shaping provided faster performance of the microcatheter position when performing aneurysm coiling embolization.

Currently, microcatheters are usually shaped by surgeons based on 3D-DSA images on a computer screen. However, 3D-DSA images lack in-depth information on vascular architecture, which makes it difficult for surgeons to obtain 3D anatomy information on aneurysms and parent arteries. Moreover, the microcatheter route is implemented according to the surgeon’s experience; therefore, several adjustments are needed before the surgeons can determine the real path of the microcatheter entering the parent artery and aneurysm sac.

Recently, 3D printing technology has been used in microcatheter shaping for endovascular treatment of intracranial aneurysms. Surgeons can shape the microcatheter according to the 3D aneurysm model before embolization, which ensures both the accuracy and stability of the microcatheter for embolization. However, 3D printing technology has some notable limitations. First, printing a solid 3D model before the procedure is costly and time-consuming, making it difficult to apply to ruptured aneurysms. Moreover, the appropriateness of the selected pathway remains uncertain since the shape of the microcatheter relies on the route imagined by endovascular surgeons according to the solid 3D aneurysm model [8,9,10,18,19]. Therefore, it may take several times to reshape the microcatheter according to 3D models, which would prolong the operation time and increase the rate of aneurysm rupture during the operation.

Compared with microcatheter shaping using 3D-printed models and conventional manual shaping processes, computer-assisted microcatheter shaping is much safer and more effective. Simulating the optimal route of the microcatheter with computer-assisted techniques during the operation just requires several minutes and makes it accessible for surgeons to shape the microcatheter in one attempt without repeats. Although the packing density of the coils and the rate of stent assist technique usage between the groups showed no significant difference, all cases achieved good clinical outcomes after embolization. This demonstrates that computer-assisted microcatheter-shaping technology can achieve the same clinical outcome as experienced operators when embolizing intracranial aneurysms. In addition, both the in vivo and the in vitro models confirmed that the speed of microcatheter positioning by computer-assisted technique is significantly faster than that by traditional methods.

Many factors can affect microcatheter shaping, such as the aneurysm anatomic characteristics, arterial paths to the aneurysms, microcatheter material, and treatment strategy for aneurysm embolization. The computer-assisted shaping technique can extract the anatomical information of the parent artery and aneurysm directly from 3D-DSA images within several minutes and then determine the most appropriate pathway. As the microcatheter material and treatment strategy of operators can also affect the microcatheter shaping, surgeons can adjust the position of the tip or support point to achieve a flexible path design. Moreover, the arbitrary resilience coefficient and shaping length can also be adjusted to ensure that the designed route is appropriate for different surgeons. Therefore, computer-assisted microcatheter shaping on 3D-DSA images will contribute to the selection of the most appropriate microcatheter route for aneurysm embolization, and this methodology would improve the effectiveness of intracranial aneurysm embolism procedures performed by inexperienced neurosurgeons and supplement their training. It should be emphasized that the traditional manual catheter-shaping technique is also a critical skill for endovascular coiling procedures. Computer-assisted microcatheter shaping is just a supplementary skill for traditional manual operator catheter shaping, which could be used as an adaptive learning tool to assist novice operators in catheter shaping. The efficacy and safety of computer-assisted techniques should be assessed by a multicenter, randomized, prospective study in the future.

## 5. Conclusions

Computer-assisted microcatheter shaping is a novel and safe method for microcatheter shaping that introduces higher accuracy in microcatheter shaping during the treatment of intracranial aneurysms. This method can be employed for improved preparation for surgery and training support.

## Figures and Tables

**Table 1:** Accuracy and delivery of microcatheter shaping in vitro.

	CAMS	MMS	*p*
Time of position(s)	114.4 ± 23.99	201.9 ± 24.54	0.015
Microcatheter delivery			0.52
Excellent	5	4	
Moderate	12	10	
Poor	3	6	

**Table 2:** Accuracy and delivery of microcatheter shaping for inexperienced and experienced doctors in vitro.

	Inexperienced Operators			Experienced Operators		
	CAMS	MMS	*p*	CAMS	MMS	*p*
Time of position(s)	93.6 ± 29.23	228.9 ± 31.27	0.005	135.1 ± 38.46	178.8 ± 37.44	0.47
Microcatheter delivery			0.5			0.82
Excellent	2	2		3	2	
Moderate	7	5		5	5	
Poor	1	3		2	3	

**Table 3:** Baseline characteristics of patients of CAMS and MMS groups.

Characteristic	CAMS	MMS	*p*
Age	54.21 ± 2.46	59.0 ± 2.02	0.139
Male (n, %)	9 (37.5)	5 (20.8)	0.204
Aneurysm size (mm)	5.13 ± 0.49	4.25 ± 0.26	0.125
Aneurysm location			1
Anterior circulation	23	23	
Posterior circulation	1	1	
Techniques			0.376
Coiling	8	11	
Stent-assisted coiling	16	13	

**Table 4:** Primary and secondary outcomes between CAMS and MMS groups in vivo.

	CAMS	MMS	*p*
Time of microcatheter position (min)	2.48 ± 0.32	5.16 ± 0.46	0.0001
Immediate Raymond classification			0.31
I	23	24	
II	1	0	
III	0	0	
mRS at discharge	0.63 ± 0.15	0.67 ± 0.17	0.85
mRS at 6 months post-op	0.46 ± 0.13	0.42 ± 0.15	0.83
Aneurysm regrowth at 6 months post-op	0	0	N/A
Accuracy			0.149
Excellent	22	24	
Moderate	2	0	
Stability			0.312
Stable	23	24	
Unstable	1	0	

Note: N/A: Not Application.

## Data Availability

The data is available from the corresponding author upon reasonable request.
